# Nanotechnologies for Physiology-Informed Drug Delivery to the Lymphatic System

**DOI:** 10.1146/annurev-bioeng-092222-034906

**Published:** 2023-03-31

**Authors:** Katharina Maisel, Claire A. McClain, Amanda Bogseth, Susan N. Thomas

**Affiliations:** 1 Fischell Department of Bioengineering, University of Maryland, College Park, Maryland, USA; 2 Wallace H. Coulter Department of Biomedical Engineering, Georgia Institute of Technology and Emory University, Atlanta, Georgia, USA; 3 George W. Woodruff School of Mechanical Engineering, Georgia Institute of Technology, Atlanta, Georgia, USA; 4 Parker H. Petit Institute of Bioengineering and Bioscience, Georgia Institute of Technology, Atlanta, Georgia, USA; 5 Winship Cancer Institute, Emory University, Atlanta, Georgia, USA

**Keywords:** drug delivery system, lymph node, bioengineering, immunoengineering, biomaterials, targeted delivery

## Abstract

Accompanying the increasing translational impact of immunotherapeutic strategies to treat and prevent disease has been a broadening interest across both bioscience and bioengineering in the lymphatic system. Herein, the lymphatic system physiology, ranging from its tissue structures to immune functions and effects, is described. Design principles and engineering approaches to analyze and manipulate this tissue system in nanoparticle-based drug delivery applications are also elaborated.

## INTRODUCTION

Lymphatic vessels connect peripheral tissues to the hundreds of lymph nodes (LNs) that are distributed throughout the body. Together, these tissues play key roles in fighting infection and maintaining tissue fluid homeostasis; appreciation for their value as drug delivery targets thus continues to increase across many disciplines. In this review, key biological features of lymphatic vessels and LNs, as well as how lymphatic transport affects immunity, are described. Established and emerging strategies to target lymphatic vessels and/or LN compartments, particularly using engineered nanotechnology-based drug delivery systems, are highlighted.

## LYMPHATIC VESSEL PHYSIOLOGY

The lymphatic system is responsible for maintaining interstitial tissue fluid homeostasis and facilitates the mounting of the adaptive immune response (1). Approximately 20 L of plasma are filtered from the blood capillaries per day, and, due to Starling’s forces, ~17 L are reabsorbed (2). The excess 3 L of fluid within the interstitial tissue space are drained into lymphatic vessels due to these vessels’ lower pressure relative to the tissue interstitium and their fluid transport functions. Once in lymphatic vessels, this fluid, which contains excess blood plasma, immune cells, antigens, cell metabolites, and foreign pathogens, is termed lymph (1). Lymph is transported unidirectionally to local draining LNs. Once in LNs, lymph that is not sampled by resident cells or filtered into the local blood capillary bed is transited out through efferent lymph vessels, eventually being returned to the systemic circulation at the thoracic duct if it is not sampled or filtered in subsequent LNs within the draining lymphatic chain.

**Figure 1:**
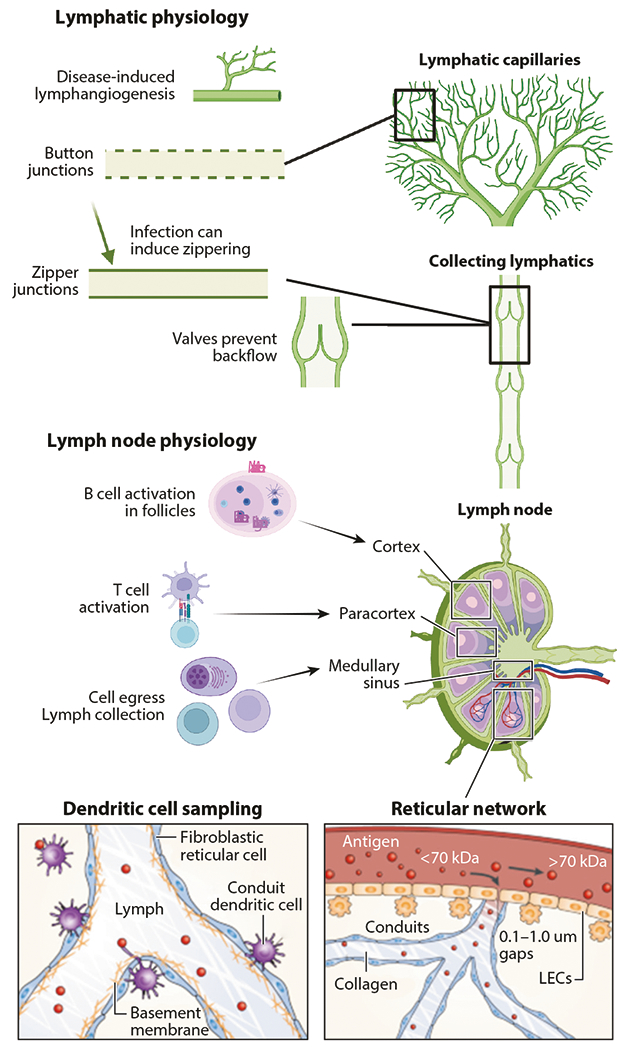
Lymphatic and lymph node physiology. Materials enter lymphatic vessels through lymphatic capillaries in part aided by button-like junctions between lymphatic endothelial cells that facilitate paracellular transport, as well as transcellular transport. Zippering can occur during disease. Collecting lymphatics are less permeable and have zipper-like junctions and valves to prevent backflow. Materials enter lymph nodes via afferent lymphatics and enter the cortex (where B cells are educated) and the paracortex (where T cells are educated) via the reticular network. Antigen-presenting cells such as dendritic cells and macrophages sample the lymph and material within the conduit system to present to T and B cells and allow for cell egress into the blood stream via high endothelial venules or lymphatics via medullary sinuses. Abbreviation: LEC, lymphatic endothelial cell. Figure adapted from images created with BioRender.com (*top, middle*) and from Reference 6 (*bottom*).

Lymphatic vessels are mainly composed of lymphatic endothelial cells (LECs) and are found throughout the body. Essentially, lymphatic vessels consist of two major distinct vessel types—initial and collecting (3, 4) (Figure 1). Both types share the same intercellular junction proteins of vascular endothelial (VE)-cadherin, zonula occludin-1, and claudin-5, but the junctions are discontinuous in initial vessels and continuous in collecting vessels (5). Other than gross structure, other features that distinguish these vessels from one another are the expression levels of lymphatic vessel endothelial hyaluronan receptor-1 (LYVE-1), which are higher for initial lymphatic vessels compared with that of collecting lymphatic vessels, and the master regulator of LEC fate, Prox-1 (4).

Initial lymphatic vessels are enmeshed within the tissue interstitium proximal to blood capillaries. They have specialized intercellular junctions that are button-like and a discontinuous basement membrane (3), features that contribute to fluid and solute uptake. LECs that compose initial lymphatic vessels resemble oak leaves in shape, with interdigitating cell flaps connected through their intermittent intercellular junctions (3). The regions of junctional protein VE-cadherin in mice are discontinuous, with approximately 3-μm gaps between junctions (3, 7). Additionally, initial lymphatic vessels have anchoring filaments that are connected to the interstitial tissue that enable sensing of interstitial fluid pressure changes associated with lymph accumulation (8). Alterations in interstitial fluid pressure result in pulling of anchoring filaments, opening cell flaps to allow drainage of interstitial fluid into the vessel. Thus, fluid and cell drainage into initial lymphatic vessels has largely been thought to be driven by passive, biomechanical processes. However, transcellular transport has also been demonstrated to be utilized by LECs (9–11): In vitro studies have demonstrated that active mechanisms, such as endocytosis, are used by LECs to transport materials, including albumin, immune cells, and nanoparticles, across the vessel wall into the lumen (10, 11).

Collecting lymphatic vessels, in contrast to initial lymphatic vessels, have intercellular junctions that are continuous, or zipper-like, and a continuous basement membrane. Surrounding the basement membrane are lymphatic muscle cells, which contract to propel lymph forward through the vessel. Collecting vessels also have luminal valves to prevent lymph backflow (12). These features enable efficient transport of lymph from the interstitial tissue site of formation to eventually be returned into circulation via the lymphatic vessel chain within its drainage basin (13).

In the intestine, lymphatics are found in the lacteals, vessels that sit within the villi structures of the intestinal tract. Facing the lumen are intestinal epithelial cells that transport fluid and nutrients toward lymphatic and blood vessels in the villi. Of note, lipids that are absorbed via intestinal epithelial cells are packaged into chylomicrons, nanoparticles ranging from 50–200 nm in size, by epithelial cells, which are transported into systemic circulation via lymphatic vessels. The process of chylomicron creation and lymphatic uptake has been described in detail elsewhere and has been taken advantage of by numerous studies modifying drugs to be processed via the normal lipid pathways (14–17). Materials absorbed by blood vessels will first be brought to the liver where they will undergo hepatic first-pass metabolism, while materials transported via lymphatics enter systemic circulation via the thoracic ducts. A detailed description of the unique aspects of intestinal lymphatic physiology and transport can be found in several excellent reviews (18–20).

Whereas lymphatic function is important in maintaining tissue homeostasis and immune signaling, disease can manifest lymphatic remodeling through a variety of mechanisms to alter the lymphatic system’s transport functions, as reviewed elsewhere (21). In brief, these include zippering after viral infection and lymphangiogenesis, the expansion of the lymphatic network, to accommodate for additional fluid load (22, 23). Lymphedema is a disease typified by swelling due to insufficient lymphatic transport. The lymphatic system and lymphangiogenesis are also implicated in cancer progression (22, 24). The opportunities and challenges these changes pose for nanotechnology-enabled drug delivery are explored in later sections.

## LYMPH NODE PHYSIOLOGY

LNs are located throughout the body, connected in chains by lymphatic vessel networks as described above. LNs are Specialized tissues enriched with lymphocytes, and the delivery of lymph via afferent vessels allows for immune surveillance of peripheral tissues. Upon entering the LN at the subcapsular sinus (SCS), lymph is disseminated through the medullary, transverse, and cortical sinus structures (Figure 1). Lymph then exits the LN via the efferent lymphatic vessels and continues flowing through the downstream lymphatic vessels, where lymph can be sampled or filtered in subsequent LNs prior to returning to systemic circulation at the thoracic duct.

Within the LN, sinus-lining cells, including LECs and macrophages, sample lymph-borne solutes, which they either process and respond to directly or transfer to other proximal cells. For example, SCS macrophages can capture and transfer large immune complexes via the Fc receptor to the basal side of the sinus where cognate B cells are located. SCS macrophages can also present nondegraded antigen to B cells at the follicular side of the SCS (25, 26) and degraded antigens to T cells (27). The reticular meshwork that forms a conduit system consisting of fibroblastic reticular cells controls lymph-borne material access to the LN paracortex in a size-dependent manner (Figure 1). Lymph-borne molecules larger than 70 kDa are largely excluded from the conduit system. However, virions larger than 70 kDa have been observed within LN conduits, a process thought to assist with rapid T cell activation (28). Molecules smaller than 70 kDa, on the other hand, are readily transited into deeper structures of the LN via the conduit system. Due to these size-exclusion barrier functions, the SCS tightly regulates the way most antigens gain access to the B cell follicle. While larger antigens are generally considered to be delivered to the B cells after capture by SCS macrophages, B cells can also directly sample and capture smaller lymph-borne antigens that diffuse into follicles from the conduits that bypass the SCS barrier. Low-molecular-weight lymph-borne macromolecules can also permeate through gaps in the SCS (0.1–1 μm) and directly access B cells in the B cell follicle (25, 29, 30). Additionally, conduits allow solute access to high endothelial venules (HEVs), structures that coordinate lymphocyte egress into LNs from the circulation and are localized deep within the LN parenchyma. These conduits provide a direct pathway for chemokines and other regulatory cues to access internal structures in the LN and rapidly regulate leukocyte infiltration into local LNs (30–32).

## IMMUNE PHYSIOLOGY OF LYMPHATIC TRANSPORT

### Lymph Protein Composition

Lymph is an aqueous fluid whose proteomic composition reflects the tissue from which it drained. Lymph composition can shape the immunological niche within the LN to promote immune tolerance, autoimmunity, or inflammation (33–39). Like blood, the most abundant proteins in lymph are albumin and globulins. However, since lymph is composed of proteins that reflect the tissue from which it drains, lymph also contains many antigens resulting from local pathogenic infections or injury, as well as tissue-specific self-antigens (33–39). Examples of proteins found in higher amounts in lymph compared with those in plasma include extracellular matrix proteins and proteins associated with tissue remodeling/growth, apoptotic proteins released from dying cells, and proteins from cellular metabolism/catabolism in the peripheral tissue from which they drain (40–45).

Much of the work identifying the lymph proteome has resulted from easy access to peripheral subcutaneous or mesenteric prenodal lymph that can be obtained during abdominal surgery or via small incisions (38). Studies have shown that lymph also contains proteins relating to intracellular processes, including histones, ribosomal proteins, and transcription factors, as well as proteins derived from organelles or cytosolic processes including cytosolic enzymes and membrane or cytoskeletal proteins (35–39). It is likely that these proteins originate either from apoptotic cells that form as part of the normal organ renewal process or from necrotic cells that appear during disease states including infections and trauma.

During inflammation and other pathogenic events, the proteins within lymph reflect not only the composition of interstitial fluid of the peripheral tissue from which it is drained but also proteomic signatures resulting from the local inflammation or pathogenic condition. For instance, in an animal model of sepsis via cecal ligation and puncture, a signature of 158 unique proteins was found in the lymph compared with that of healthy animals (46). This included an increase in proteins involved in lipid metabolism, specifically apolipoprotein E, annexin A1, neutrophil gelatinase-associated lipocalin, S100a8, and S100a9. These proteins appeared to be associated with disease progression and could suggest that specific protein signatures within lymph could be used as biomarkers for disease detection and progression. In another study, lymph collected from patients following abdominal trauma showed an increase in tissue-specific proteins and damage-associated molecular pattern response such as mediators of the acute inflammatory response, proinflammatory molecules, and vasculogenic proteins, all of which are classically associated with tissue trauma (40, 41, 47, 48).

New evidence has demonstrated that peripheral lymph not only contains proteins but also is crucial in transport and dissemination of extracellular vesicles from peripheral tissues (49–52). Furthermore, this emerging body of work suggests that lymph-transported extracellular vesicles may contribute to forming the specific immunological niche within the draining LNs. Researchers showed that lymphatic exudate from melanoma patients was enriched with microRNAs usually associated with extracellular vesicles (49, 50). Additionally, they demonstrated that tumor-derived extracellular vesicles are taken up and primarily transported by lymphatic vessels. Extracellular vesicles derived from tumors have been shown to be transported via the lymphatic system and can enhance LN metastasis through shaping of the premetastatic niche that enhances lymphangiogenesis and tumor cell adhesion via induction of vascular cell adhesion molecule (VCAM)-1 expression on LECs (53, 54). Extracellular vesicles have also been shown to be elevated in lymph in various inflammatory conditions including atherosclerosis and rheumatoid arthritis (51, 52). Distributing peripheral tissue–derived extracellular vesicles to LNs and into the systemic circulation is a crucial role the lymphatic system thus plays.

### Immune Cell Migration

In addition to their roles in fluid transport, lymphatics are known for transporting immune cells from peripheral tissues to LNs. In particular, antigen-presenting cells (APCs) migrate from infected, inflamed, or damaged tissues into lymphatic vessels, often through chemotaxis via CCR7/CCL21-, CCL19-, or CXCR3/CXCL12-regulated axes. Immune cells enter lymphatic capillaries and initially migrate along the vessel wall downstream until they reach collecting lymphatic vessels. Within collecting vessels, these cells are then convectively transported with lymph fluid until they reach LNs. In the LNs they may cross the LEC barrier on the inferior side of the SCS to enter the LN cortex and paracortex. Two recent key reviews highlight the various aspects of lymphocyte migration within lymphatics and LNs and into the systemic circulation (55, 56).

### Lymphatic Transport Affects Immunity

Lymphatic transport to LNs underlies how adaptive immune responses or tolerance to peripheral antigens are initiated and regulated. For example, in a mouse model with missing dermal lymphatics, due to expression of soluble vascular endothelial growth factor (VEGF) receptor 3 (VEGFR3) downstream of the keratin promoter (K14-VEGFR3-Ig) (57), antibody responses to vaccination were drastically reduced and the T cell response was delayed (58). However, skin autoimmunity phenotypes were also present in K14-VEGFR3-Ig animals of advanced (>1 year) age, suggesting that lymphatic transport is crucial for maintaining immune tolerance. Cutaneous infection with vaccinia virus has been shown to induce lymphatic capillary junction tightening, or zippering, to reduce fluid transport and prevent dissemination of the virus in a VEGFR2-dependent manner (59). In the absence of lymphatic vessels, antiviral immunity was impaired and viral clearance was delayed, in part through incomplete responses in the LNs (57). Similarly, lymphatic capillaries exhibit zippering during mycoplasma pulmonis infection of the trachea, which is thought to reduce transport of fluid and dissemination of infectious materials (7). Zippering can also occur in intestinal lacteals in response to removal of the local microbiome via antibiotics (60) or through increased VEGFA levels (61), consistent with the work on vaccinia infection that suggests that VEGFR2 signaling is responsible for lymphatic zippering. These results suggest that lymphatic transport is crucial for the induction of a robust adaptive immune response and that lymphatics have evolved to prevent further dissemination of pathogens like viruses from the infected site through tightening their junctions in initial vessels.

Disease models with altered lymphatic transport offer the opportunity to determine these alterations’ effects on adaptive immune response. It is difficult to assess what altered transport, however, since APC presence, lymph drainage of antigen and cytokines, fluid flow, and so on can all affect lymphatic transport functions. To distinguish the role of the former two processes in the development of adaptive immune response and the effects of disease on these processes, we recently leveraged a synthetic antigen system wherein peptide antigens were covalently linked using a reversible linker to nanocarriers that, due to their size, are restricted in their lymphatic vessel access and transport to LNs via two discrete mechanisms, migrating APCs and lymph drainage (see discussion on design parameters governing nanocarrier delivery profiles in subsequent sections). Responses by endogenous lymphocyte populations or donor T cells that recognize the exogenously administered antigen were then compared. Strikingly, while antigen that was delivered to LNs by migrating APCs resulted in a greater expansion of CD8 T cells with cytotoxic functions, lymph-draining antigen instead resulted in a much greater expansion of the pool of stem-like CD8 T cells in LNs (62). CD8 T cell immunity elicited by either transport process, however, increased infiltration of effector-like CD8 T cells and improved tumor control. These results suggest that not only the resulting quantity but also the resulting quality of CD8 T cell immunity that is elicited in LNs mediated by lymphatic transport is compartmentalized by route of antigen transport, results that imply how the relative effects of altered lymphatic transport on APC migration versus lymph drainage may play a role in fine-tuning the resulting adaptive immune response.

As seen in skin, impaired lymphatic transport has been shown to occur in intestinal inflammation, where lymphangiogenesis often accompanies chronic inflammation. Recent work has demonstrated that tertiary lymphoid organs form in association with lymphatic vessels and can restrict intestinal lymphatic drainage and immune cell migration in Crohn’s disease, a subset of inflammatory bowel disease (63, 64). Tertiary lymphoid organs were found near collecting lymphatic vessels close to valve regions and halted immune cell trafficking to draining LNs. The impaired drainage appeared tumor necrosis factor (TNF) dependent. Furthermore, TNF stimulation prevented LECs from expressing valve-associated genes, suggesting that loss of valve integrity has a role in impairing normal lymphatic transport in ileitis. It was hypothesized that this disrupted lymphatic transport and immune trafficking contributes to the chronic intestinal inflammation in Crohn’s disease.

The role of main lymphangiogenic pathway VEGF-C/VEGFR3 has been experimentally explored using anti- and prolymphangiogenic therapies such as blocking antibodies or recombinant VEGF-C. The collective body of work suggests that blocking lymphangiogenesis enhances inflammation in disease models including irritable bowel syndrome, arthritis, allergies, and skin inflammation (65–70). In contrast, treatments that enhance lymphangiogenesis lead to enhancement of lymphatic transport and reduced disease severity, as demonstrated by reduced inflammation in irritable bowel syndrome and chronic skin inflammation (71, 72). We point readers to two recent reviews for a more comprehensive discussion (73, 74).

## NANOTECHNOLOGY-ENABLED LYMPHATIC VESSEL TARGETING

**Figure 2:**
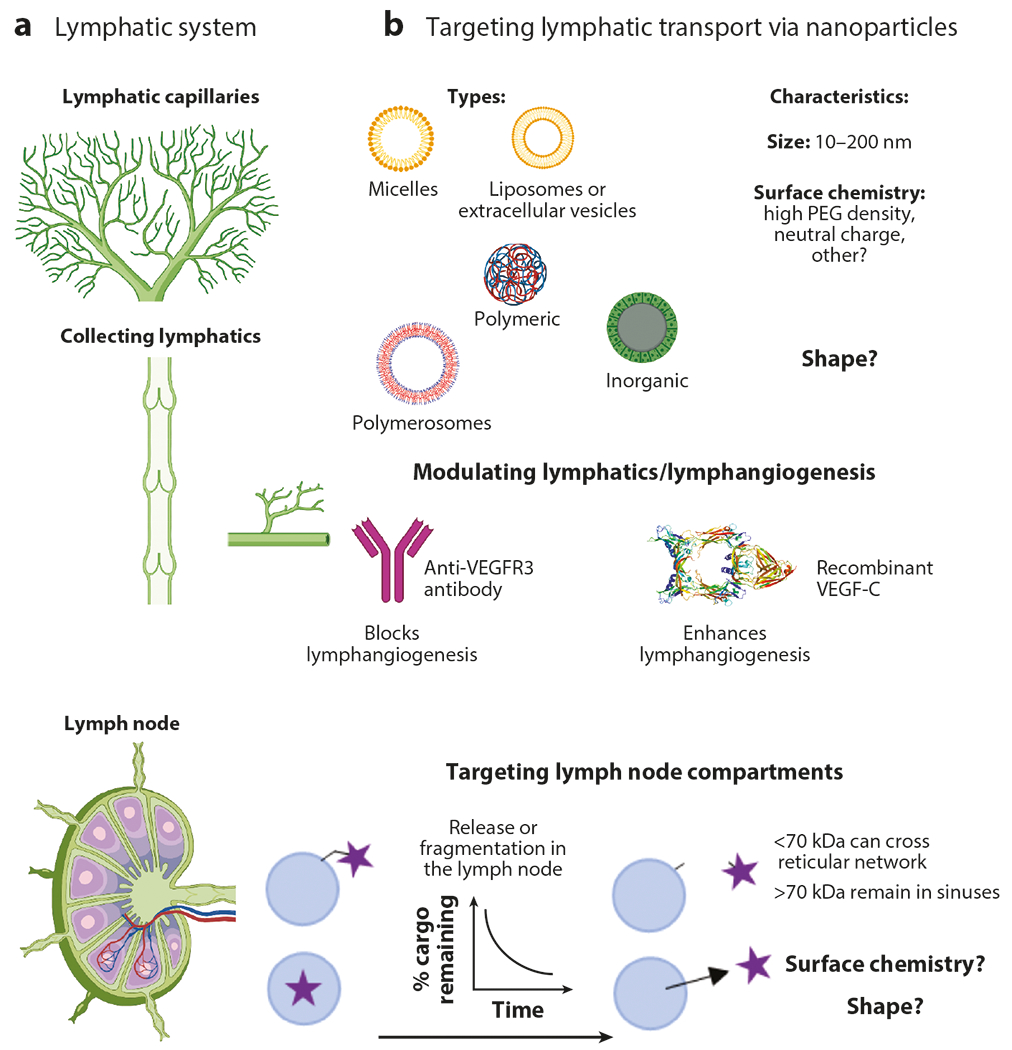
(*a*) Lymphatic system including initial lymphatics, collecting lymphatics, and lymph nodes. (*b*) Strategies for targeting lymphatic vessels and lymph nodes. Nanoparticles between 10 and 200 nm in size are transported into lymphatic vessels, and transport is optimized through dense PEG coatings and neutral charge (*top*) (11, 12, 14, 133). Lymphatic functions can be modulated through blocking or enhancing lymphangiogenesis (*middle*) (53, 58, 65, 67, 68, 70, 72, 86, 87, 120). In the lymph node, materials <70 kDa in size can cross into the reticular network, while larger molecules stay within the sinuses (*bottom right*). Therapeutic delivery to the cortex and paracortex can be achieved through targeting nanoparticles to the sinuses that release small cargo mediated through diffusion or chemical release (*bottom left*) (76, 92–94). Abbreviations: PEG,poly(ethylene glycol); VEGF, vascular endothelial growth factor. Figure adapted from images created with BioRender.com.

Nanoparticle targeting to the lymphatic system has been of substantial interest in the past two decades. A number of particle formulations have been developed to target lymphatic vessels, which lead to entry to specialized areas of the body, including LNs or brain tissue, to elicit a therapeutic effect. Nanocarrier size is well described to be a characteristic regulating nanoparticle transport into lymphatic vessels. Nanoparticles <10 nm are poorly retained in the tissue interstitium due to their high diffusivities and the permeability of the blood capillaries (75). Nanoparticles >200 nm, on the other hand, are largely restricted within the tissue site of injection due to the fact that they are larger in hydrodynamic size than the local matrix porosity and, as a result, generally only enter lymphatic vessels and are trafficked to LNs by virtue of the phagocytic and migratory functions of APCs patrolling the peripheral tissue site (76). Nanoparticles that are retained at the tissue injection site as a result of their inability to freely traverse the blood capillaries but are small enough to transit the pore size of the local extracellular matrix mesh, roughly between 10 and 200 nm in hydrodynamic size, exhibit behaviors of robust interstitial transport and lymphatic uptake and are thus considered optimal for lymphatic drag delivery achieved by injection into a peripheral tissue, such as the skin, subcutaneous tissue, fat pad, or muscle (Figure 2). We recently demonstrated that both size and surface chemistry are crucial for nanoparticle transport into lymphatic vessels. We found that grafting of poly(ethylene glycol) (PEG) to the surface of a nanoparticle can be used to improve lymphatic uptake (11). Transport of 100- and 40-nm PEGylated polystyrene nanoparticles was enhanced across an in vitro model of LEC uptake compared with that of uncoated nanoparticles. Specifically, an R_f_ /D > 4 provided the highest transport efficiency in vitro, effects that were recapitulated in vivo. Additionally, PEGylated nanoparticles with PEG coatings with an R_f_/D > 4 and a neutral charge had maximal transport across LECs in vitro compared with that of positively or negatively charged nanoparticles, and both paracellular and transcellular transport mechanisms were involved. Neutrally charged and densely PEGylated nanoparticles (R_f_/D > 4) had longer transport distances and higher LN accumulation compared with those of uncoated particles. Overall, our work demonstrated that PEG density is an important consideration that modulates nanoparticle transport into lymphatic vessels. For further considerations in design criteria for lymphatic drug delivery, we point the readers to a recent review (77).

Salient to the concept that nanotechnology uniquely enables drug targeting to lymphatic vessels themselves, our group recently described a lymph-draining nanoparticle formulation that realized the lymphatic function modulating effects of a small-molecule L-type calcium activator in vivo. In this work, Bay K8644 formulated into poly(propylene sulfide) nanoparticles (PPS NPs) 30 nm in hydrodynamic size, which drain into lymph after injection in the skin, restored pump function in a murine model of lymphedema, while simultaneously reducing the agent’s dose-limiting side effects (78). This was the first demonstration, to our knowledge, of direct lymphatic vessel targeting achieved by a nanocarrier to modulate lymphatic vessel function itself.

Oral nanoparticle delivery to intestinal lymphatics is also of high interest due to its potential to either avoid hepatic first-pass metabolism or treat intestinal diseases. Several examples exist of lipid-based nanoparticle formulations aiming to hijack lipid metabolism pathways that transport lipids from the lumen of the intestine into the underlying lymphatics. In one example, 190-nm solid lipid nanoparticles containing the antiretroviral drug atazanavir sulfate were used to improve drug transport to Caco-2 enterocyte-like cells in vitro (79). In vivo, this formulation resulted in improved drug bioavailability in draining LNs. Uptake was lymphatic dependent, as drug levels in blood were reduced with lymphatic uptake blocking cycloheximide treatment. A glyceryl behenate lipid formulation loaded with the drug genistein was also used to form chylomicron-like structures for transport across the intestinal epithelium (80). Incubation of this nanoparticle system on enterocyte-like cells in vitro with phosphatidylcholine and/or cholesterol increased their diameter. The nanoparticle system contained multiple nucleated vesicles on the surface, indicating a chylomicron-like structure. Nanoparticle uptake in porcine duodenum ex vivo occurred within 1–2 h after administration, suggestive of a chylomicron uptake pathway. Solid lipid nanoparticles (SLNs) and nanostructured lipid carrier formulation of 6-methoxyflavone have been found to result in preferential uptake by Caco-2 cells, increasing the C_max_ of the drug in the mesenteric LNs after oral administration. Both nanoparticle formulations enhanced bioavailability of 6-methoxyflavone nanoparticles, hypothesized to result from particle-mediated transport via lymphatic vessels.

Another strategy to target chylomicron pathways is to use bile acid coatings. In one study, 194-nm liposomes loaded with insulin were coated with chondroitin sulfate-*g*-taurocholic acid that provided higher oral bioavailability compared with that of uncoated liposomes and a sustained decrease of blood glucose levels 16 h post administration (81). A similar nanoparticle was designed using glycocholic acid conjugated SLN (82), with surface modification increasing particle uptake in a breast cancer cell line known to express an apical sodium-dependent bile acid transporter also found in the intestine. Glycocholic acid density and nanoparticle size affected the bioavailability significantly: Optimal bioavailability of 47% occurred with a 40% surface coating with glycocholic acid on 100-nm nanoparticles. In a continuation study, food intake immediately after oral administration of glycocholic acid conjugated solid nanoparticles resulted in significantly lower bioavailability (83). Feeding prior to administration also decreased bioavailability, and optimal bioavailability resulted from a fasting time of 4 h before and 30 min after administration. Oral administration of glycocholic acid conjugated solid nanoparticles resulted in nanoparticle recovery in both plasma and lymph, suggesting lymphatic uptake. A glycocholic acid–chondroitin sulfate coating of 120-nm SLNs loaded with the common chemotherapy drug docetaxel (84) increased drug bioavailability. These data suggest that bile acids can be used to lead nanoparticles to be transported across the intestinal epithelium via chylomicron pathways, resulting in their transport to intestinal LNs and into systemic circulation via lymphatic vessels.

A challenge with oral drug delivery is avoiding burst release of the drug into the stomach due to the low pH environment. A modified chitosan coating, specifically N-carboxymethyl chitosan, was recently shown to limit gastric release of curcumin and increase lymphatic uptake of the drug (85). This coating does not degrade at low stomach-like pH but will disintegrate at pH > 5, releasing the drug. After oral administration to rats, N-carboxymethyl chitosan-coated nanoparticles had the highest bioavailability. LN accumulation of curcumin at 4 h after oral administration was also higher using N-carboxymethyl chitosan-coated nanoparticles compared with that of chitosan-coated nanoparticles. Overall, N-carboxymethyl chitosan coatings improve nanoparticle-mediated curcumin delivery to the lymphatic system compared with that of free curcumin solution or unmodified chitosan, indicating that the surface chemistry of the nanoparticles is important not only for stabilization of drugs in the harsh environment of the stomach but also for delivery into the intestinal lymphatics.

A recent two-part study assessed the effect of size and surface charge on lymphatic transport of glyceryl behenate solid nanoparticles carrying the antilymphangioleiomyomatosis drug rapamycin (86, 87). Nanoparticles 200, 500, and 1,000 nm in size were assessed on the basis of their ability to transport across the respiratory epithelium and the lymphatic epithelium, to inhibit proliferation of TSC2-negative mouse embryonic fibroblasts, and to inhibit lymphangiogenesis. In this study, 200-nm nanoparticles had the most effective transport across epithelial barriers and reduction of growth of TSC2-negative mouse embryonic fibroblasts. In addition, coating these nanoparticles with hexadecyltrimethylammonium bromide, making them highly negatively charged, enhanced these effects and also significantly inhibited lymphangiogenesis. Overall, this study showed that surface charge can modulate the ability of nanoparticles to cross cellular membranes, resulting in various treatment efficacies.

Lymphatic vessels around and throughout the brain have recently received more interest in the field of targeted drug delivery. A photodynamic therapy agent, indocyanine green (ICG), was loaded into poly(lactic-co-glycolic acid) (PLGA) nanoparticles to test the effect of treating glioblastoma via subcutaneous injection in the neck of mice (88). Subcutaneous injection of 45-nm nanoparticles led to the highest accumulation of ICG in the brain parenchyma when compared with that of 113- and 181-nm nanoparticles and ICG. This suggested that the mechanism of transport to the brain was via the lymphatic system and in part was due to immune cell trafficking. In vivo treatment efficacy was assessed in an orthotopic glioblastoma C6 mouse model with ICG. Nanoparticle treatment provided tumor suppression for 41 days and a 50% higher survival rate after 45 days compared with that of free ICG after subcutaneous, nanoparticle, or free ICG injection intravenously. Thus, the nanoparticle delivery of ICG through the lymphatic vessels provided higher accumulation in the brain and enhanced photodynamic treatment. Overall, designing nanoparticles that can take advantage of the innate brain lymphatic vasculature was shown to be a useful route for enhancing the bioavailability of drugs unable to cross the blood–brain barrier.

Biological materials have also been used as a coating to target nanoparticles to lymphatic tissues as glioma treatment. For instance, *Saccharomyces cerevisiae* yeast capsules are composed of β-glucans, which can be used as an active target that binds to dectin-1 that is expressed on magnocellular cells in the brain. β-Glucans were conjugated to temozolomide, a common glioma therapeutic, via disulfide bonds and formulated into 74-nm nanoparticles (89). This therapeutic relied upon hitchhiking on macrophages to bypass the blood-brain barrier via lymphatic vessels to reach brain tumors. At these sites, there is an increased level of glutathione, which cleaves disulfide bonds, releasing the antitumor drug. The nanoparticle system was successfully transported across magnocellular cells and phagocytosed into resident macrophages. Overall, this nanoparticle had higher antitumor efficacy with higher survival rates, higher elimination of tumor cells, and higher rates of apoptotic cells than those of control groups. This technology demonstrated the use of natural target of β-glucan on yeast capsules that used lymphatic transport and macrophage hitchhiking to overcome the blood–brain barrier to treat gliomas.

Nanoparticles have also been developed to transport contrast agents used for magnetic resonance imaging to enhance visualization of the lymphatic system. Traditional contrast agents are administered via intravenous injection, which results in low specificity and sensitivity to visualize lymphatic vessel structures. Recently, a calcium phosphate nanoparticle coated with PEG-alendronate and loaded with a contrast agent was developed to test its enhancement of lymphatic system visualization under magnetic resonance imaging (MRI) (90). They found that contrast efficiency of the nanoparticle formulation was 1.6-fold higher than that of the control free contrast agent solution. Additionally, contrast agent accumulated in popliteal LNs within 20 min and up to at least 50 min after subcutaneous injection, with a peak intensity at 40 min. Free contrast agent injection produced significant variability in the signal, and the signal did not consistently appear. This was interpreted to mean that the nanoparticle formulation led to more reproducible and higher contrast in LNs, suggesting that these formulations are better for MRI visualization.

Assessing how nanoparticles affect lymphatic transport functions is difficult, since lymphatic vessels are not easily visualized and are often buried deep within tissues. To mitigate this, an ex vivo perfusion system of a lymphatic vessel was developed and used to assess the effects of nanoparticles on lymphatic vessel contractility and cell health (91). The researchers isolated the lymphatic vessel from a rat, cannulated it to two glass pipettes, and assessed the response to acetylcholine and thromboxane A_2_ (U-46619) using nanoparticles made from carbon nanohorns, multiwalled carbon nanotubes, and silver nanoparticles. The system also included extraluminal fluid to simulate interstitial fluid flow. With constant hydrostatic pressure, the lymphatic vessel spontaneously contracted, and acetylcholine and U-46619 halted the spontaneous contractions and induced sustained relaxation or contraction, respectively. When testing the carbon nanohorns, transient changes in the spontaneous contraction were observed, with a higher concentration irregularly halting contractions and causing abnormal vessel contraction, while a lower concentration did not affect contraction. When silver nanoparticles of low and high concentrations were perfused into the vessel lumen, the contractions halted after 3–4 minutes and endothelial cells appeared damaged, suggesting that silver may be toxic to the endothelium. Overall, this system provided a tool to study nanoparticle effects on lymphatic vessels and on endothelial cells, including validating the safety of the nanoparticle systems. Future research using this technology can be conducted to determine what nanoparticle types and properties negatively affect LECs and lymphatic vessel functioning, to improve nanoparticle design.

## NANOTECHNOLOGY-ENABLED LYMPH NODE DRUG TARGETING

The LN structures that tightly regulate solute transport implicated in the regulation of immune signaling likewise influence the distribution of therapeutics and engineered drug delivery systems to LN resident leukocytes. This section summarizes the consensus of principles governing how therapeutics have been engineered to gain access to and exert effects on LN cells.

Nanocarriers have been widely explored for their potential to mediate delivery to LNs. This is due to their favorable behaviors with respect to enriched lymphatic uptake when formulated at an ultrasmall scale (10–200 nm in hydrodynamic size, as discussed previously), along with the general benefits of realizing drug delivery systems in the context of LN drug delivery applications. These benefits include, but are not limited to, high amounts of drugs to be delivered in a single payload, multiple agents with synergistic activities to be delivered to a single cell, and triggered release for controlled delivery.

Like those in the peripheral tissues, LN APCs are highly efficient at taking up particles, making nanoparticles highly effective in accessing these potent APCs (Figure 2). However, both size and material composition additionally influence nanoparticle association by LN APCs (76, 92, 93). This is because in addition to regulating lymphatic access, hydrodynamic size influences intra-LN transport, with smaller particles generally exhibiting greater access to the LN parenchyma. So, whereas the extent and rate of nanoparticle drainage to and accumulation within the LN is inversely correlated with hydrodynamic size, smaller nanoparticles generally exhibit the highest levels of association with LN dendritic cells (DCs) that are more distal from the sinus (62, 92). In addition to access, nanoparticle clearance, which has been shown to be regulated by hydrodynamic size, influences particle association levels with LN cells. This has been specifically explored in the context of follicular DCs, resident stromal cells that form networks located in B cell follicles and can acquire and retain antigens for months (26). Follicular DCs clear small particles (5–15 nm) after 48 h through endolysosomal escape or extracellular vesicles. However, larger particles (50–100 nm) persist for more than 5 weeks. As a result, larger nanoparticles (50–100 nm) exhibit dramatically higher (175-fold) delivery of antigen to follicular DCs and are associated with fivefold enhancements in both germinal center B cell formation and antibody production compared with those of nanoparticles of smaller sizes. Thus, nanoparticle size that tunes humoral immunity and vaccine efficacy (26) influences association with LN APCs by regulating their APC access and mechanism of clearance.

Due to the barrier functions of the SCS, numerous nanoparticles exhibit a high propensity for accumulation in LNs after administration in peripheral tissues due to their lymph-draining capabilities. Targeting of cell populations that reside within the LN parenchyma, especially those that are lowly phagocytic, with these same nanoparticles tends to be poor (94). To overcome this limitation, a multistage delivery approach has been described, wherein lymph-draining nanocarriers are leveraged to gain access to lymphatic vessels in combination with linkers that degrade prior to nanocarrier uptake by local cells (95, 96). One such formulation includes oxanorbornadienes that undergo retro Diels–Alders fragmentation in a chemical microenvironment-independent fashion in combination with either PPS NPs or virus-like particles. Using such a system, a >100 times increase in cargo delivery to T and B lymphocytes in LNs was achieved compared with levels seen for free small-molecule cargo, which had low extents of lymphatic uptake due to clearance into blood from the injection site, and the nanocarriers themselves, which were restricted to the LN periphery and the local phagocytic populations that sample lymph from the LN sinuses. This concept extends to cargos that are directed into lymph by conjugation chemistries that are reversible in the extracellular milieu, as well as those that are passively encapsulated, so long as the release can occur in lymph soon after NP uptake into lymphatic vessels (95, 96). Due to the enhanced extent of delivery, this approach can thus benefit applications wherein the effects of immunomodulatory or cytotoxic agents should be elicited against lymphocyte populations that reside in these restricted areas of LNs. For example, these systems could be of benefit for the treatment of immune disorders or LN lymphomas or micrometastases. This approach also offers interesting opportunities for sequential delivery of agents into LNs from a single injection, as the timing of cargo association with parenchyma-resident cells corresponded with linker half-life (95). Examples may include antigens and adjuvants for vaccines, and chemoimmunotherapy combinations. Similar approaches to target and locally release drugs over time in the LNs have also been explored in the context of direct LN injections of microparticle depots, though these do not take advantage of lymphatic transport (97).

Surface modification of nanoparticles can also enhance targeting to specific cell types within LNs. For example, to target LN DCs, nanoparticles 10–50 nm in diameter have been engineered through self-assembly of phospholipids and antigen-containing fusion peptides. Through the effects of both their size and surface modification, nanoparticle uptake can be highly skewed toward DCs and macrophages (98). While DCs have a natural strong phagocytic capacity, mature DCs have reduced capacity for nonspecific endocytotic uptake (99) compared with that of immature DCs (100). To overcome this, particles were modified with α-helical peptide that strongly targets scavenger receptor class B1 (101, 102), increasing the efficiency of particle uptake by mature DCs compared with that by immature DCs due to differences in scavenger receptor class B1 expression between the two and resulting in improved targeting of mature DCs and cytotoxic CD8 T cell activation when used as a vaccine carrier (98).

Similarly, incorporating targeting antibodies to specific APC populations is another way to enhance cellular uptake and increase cargo retention within the LNs. For instance, modification with specific monoclonal antibodies targeting CD40, DEC-205, and CD11c increases uptake of pegylated PLGA nanoparticles encapsulating model protein antigen ovalbumin (OVA) by DCs. When used in vaccination applications, no differences in CD8 T cell immune response between the different targeting moieties were noted. However, all of the targeted NPs elicited enhanced CD8 T cell responses and targeted cell lysis compared with those of untargeted nanoparticles (103), demonstrating the utility of the approach.

Targeting antibodies have also been used to direct nanoparticles carrying therapeutic cargo via administration into the systemic circulation (e.g., blood) to LN HEVs to achieve delivery of therapeutic cargo to LNs. Specifically, PLGA microparticles functionalized with the targeting antibody MECA-79, which binds to peripheral node addressin, accumulated in LNs to greater extents compared with those of unmodified particles. These effects were demonstrated to be target specific, as administration of peripheral LN addressin targeting antibody reduced microparticle accumulation in LNs to levels seen for unmodified particles. Using this system, the authors found that the effects of the immunosuppressive drug tacrolimus in induction of skin allograft acceptance were augmented (104). An antibody-nanoparticle conjugate system has likewise been used to target LN T cells for immunomodulation. Conjugation of antibodies targeting T cell surface-expressed CD3 enhanced the delivery to LN-resident lymphocytes of locoregionally administered 30-nm PPS NPs that, due to their ultrasmall size, are optimal for lymphatic uptake and LN accumulation. Extending this concept to antibodies against immune checkpoints as both a targeting moiety and signal-blocking therapeutic, targeting of PPS NPs improved therapeutic synergies of PPS NP–encapsulated small-molecule immunomodulators with immune checkpoint targeting antibodies to slow tumor growth and prolong animal survival (105).

Beyond nanocarriers, multiple protein or macromolecule engineering strategies have been devised to enable improved delivery of payloads to LN cells. This includes the incorporation of ligands that target phagocytic APC populations to enhance cellular uptake of therapeutic cargo and additionally increase retention within targeted LNs. An example is the incorporation of the protein OVA into an antibody targeting the endocytic receptor DEC-205, which is abundant on these cells in lymphoid tissues, through covalent cross-linking (106). When administered in conjunction with agonistic antibody targeting CD40, a single low dose of the antibody-conjugated antigen resulted in improved elicitation of CD4 and CD8 immunity compared with that of soluble OVA delivered with complete Freund’s adjuvant or CD40, which both induce potent CD8 immunity (106). Engineering protein retention is another approach that has been explored to modulate LN delivery and the effects of cytokine therapies. For example, a fusion protein of albumin and interleukin (IL)-4, when administered systemically in a mouse experimental autoimmune encephalomyelitis model, improved disease prevention compared with that of IL-4 alone (107). These improvements were associated with fusion protein accumulation in the draining LN as well as a locally immunosuppressed environment of the LN.

Migratory immune cells such as DCs and T cells that have the natural ability to home to LNs have been exploited to achieve LN delivery of cargos. In so doing, the native or engineered migratory functions of these cells can be leveraged, offering tight control. However, the complexities of loading cells ex vivo prior to transfer, or seeking to “backpack” by targeting in situ, offer challenges with respect to selectivity, off-target effects, and magnitude of on-target delivery. For instance, large particles unable to drain into lymph directly after subcutaneous, intradermal, or intramuscular injection can be taken up by tissue-resident DCs that then travel to the LN, draining the tissue injection site (58, 62, 93, 96). This is a widely explored approach for particulate-based vaccine formulations (93). Micelles co-delivering OVA antigen with plasmid DNA encoding CCR7 have also been used to encourage DC migration to LNs through upregulation of CCR7 by DCs transfected with the plasmid at the local injection site, resulting in increased CD8 T cell priming that improves tumor control (108). Alternatively, ex vivo conditioned DCs widely studied for vaccine applications are known to home to LNs after administration into the circulation or locoregionally (108) and have been extensively investigated for therapeutic applications both preclinically and in clinical settings. Backpacking drug carriers onto circulating T cells is another strategy that has been leveraged as a mechanism to achieve LN delivery through these cells’ native LN homing mechanisms (109). For example, PLGA microparticles functionalized with antibodies targeting CD8 that were administered intravenously increased and sustained their accumulation in LNs over 24–48 h, whereas their tumor accumulation diminished precipitously over this time frame. When targeted to programmed cell death 1 (PD-1), synergies with particle-incorporated small-molecule immunomodulators were augmented (109). The antibody-nanoparticle conjugate system based on ultrasmall PPS NPs likewise accumulates in LNs after intravenous administration when targeted to T cell surface-expressed markers (105). PD-1 targeting of NPs results in improved synergies of an NP-encapsulated small-molecule immunomodulator in the treatment of LN metastasis, improving survival in an in vivo breast tumor model (105).

## INTERSTITIAL TISSUE EFFECTS ON DELIVERY OF NANOFORMULATIONS TO LYMPHATIC VESSELS

**Figure 3:**
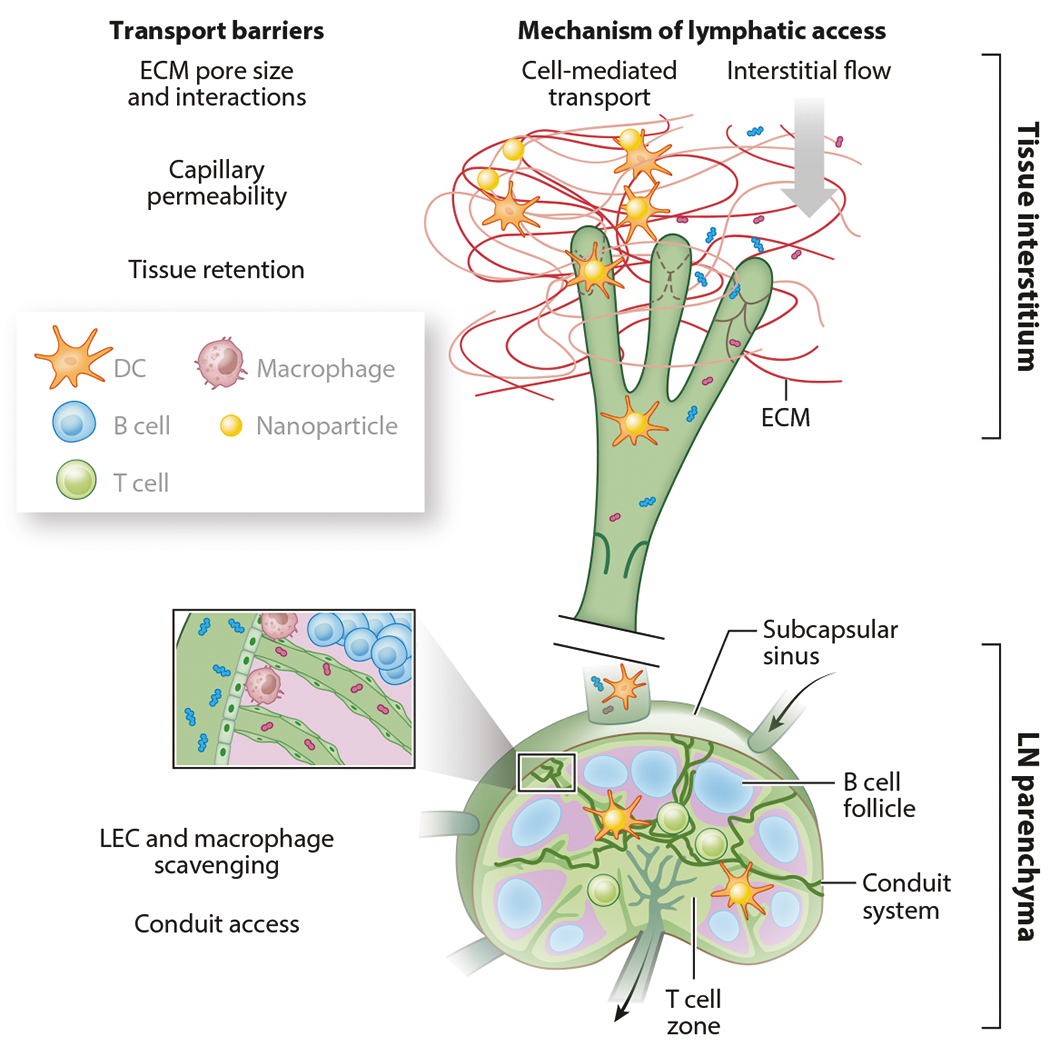
Lymphatic tissue transport barriers’ influence on access by LN resident cells to lymph-derived molecules and particles. Abbreviations: DC, dendritic cell; ECM, extracellular matrix; LEC, lymphatic endothelial cell; LN, lymph node. Figure adapted with permission from Reference 96.

To reach lymphatic vessels, and through them the downstream LNs, nanoparticles must first cross the interstitial tissue made up of the extracellular matrix and stromal cells (Figure 3). A large body of work has focused on characterizing the composition of these spaces in various tissue compartments (110), but the physical structure within the tissue compartments, particularly a complete picture that includes cells, is less understood. Many extracellular matrix materials are charged and provide ligands for cells that could also form charge-based or hydrogen bonds with nanoparticles traversing this space. We and others have found that the surface chemistry of nanoparticles can significantly affect their ability to traverse the extracellular matrix and thus is an important under-studied consideration (111, 112). Additionally, extracellular matrix remodeling can occur during diseases and inflammation, including fibrosis, causing deposition of the extracellular matrix, and edema, causing swelling and fluid accumulation in interstitial tissues (110). Such remodeling can modulate the interstitial tissue spacing and properties of the extracellular tissue and can alter how well nanoparticles are transported through the interstitium and toward lymphatic vessels. The known size limitations for lymphatic transport of nanoparticles have been largely found empirically, and further understanding about how these are altered during disease is still needed. Similarly, understanding of the factors (beyond size) influencing nanoparticle transport across LECs, including nanomaterial properties such as shape and surface chemistry, is still limited and requires further investigation.

## EFFECTS OF LYMPH NODE REMODELING ON NANOPARTICLE-ENABLED DRUG DELIVERY

LNs are highly complex secondary lymphoid organs whose structure plays pivotal roles in shaping the locally elicited adaptive immune response. Underscoring this point is the substantial remodeling of the LN’s extracellular matrix (113), fibroblast reticular cell meshwork (114, 115), and vasculature during immune challenge. LNs reacting to immune challenge also exhibit substantial changes in their biophysical properties, including their size, cellularity stiffness, and matrix composition (32), that may influence the local distribution of immune cells as well as cues that direct cell migration and shape immune responses (Figure 3). LN structures and biophysical properties are also disrupted in states of disease, such as cancer (116), inflammation (117), and infection (118, 119), among others (120), and such changes may alter the LN’s capacity to coordinate an effective immune response, resulting in pathological immune dysregulation.

One such example is tumor-draining LNs (TdLNs), which receive lymph-borne factors derived from the tumor and are widely appreciated to undergo substantial remodeling during disease progression. Changes include overall tissue enlargement and increased tissue stiffness (121) and deposition of extracellular matrix components, in addition to alterations in intranodal fluid pressures (121–124). The LN stroma also remodels, with fibroblastic reticular cells proliferating in response to tumor-derived signals, resulting in matrix remodeling and altered chemokine and/or cytokine signaling, which affects immune cell recruitment, migration, and activation (125). These factors may alter lymphocyte circulation within the LN and thus the ability to coordinate an effective immune response. With tumor progression, the functions of CD169^+^ macrophages that line the SCS and scavenge extracellular vesicles secreted by tumors that accumulate within TdLNs are also compromised, allowing vesicles to gain deeper access to the LN and interact with B cells to initiate tumor-promoting humoral immunity (126). How they influence drug delivery, let alone adaptive immune response, which has been more vigorously investigated in the literature to date, is only beginning to emerge. Initial studies in a preclinical melanoma model revealed that access to lymph-borne solutes was altered in TdLNs (62). This was achieved by using a panel of fluorescent tracers that span a range of hydrodynamic sizes over which transport via passive lymph drainage, versus uptake by migratory APCs, predominates. Patterns of tracer association with LN leukocytes when coadministered locoregionally into the naive skin or a melanoma were then compared between disease states (tumor-naive versus tumor-bearing). Lymph-draining tracers administered into a melanoma tumor accumulated in TdLNs at lower levels overall compared with tracers administered in the skin of naive animals, but they associated with LN B cells to equivalent extents (11). 30-nm tracers also associated with plasmacytoid DCs at increased levels in TdLNs compared with those in LNs of naive animals (62). Presentation of antigen delivered into lymph by tethering to PPS NPs 30 nm in hydrodynamic size by LN cells was also sustained across disease states (62). This suggests that while lymph drainage may be reduced overall as a result of disease, the LN remodels to sustain or increase access by phagocytes within the LN parenchyma. This is consistent with the increased permeability of the SCS of TdLNs in melanoma (62, 125). Interestingly, these analyses also revealed that migration by APCs to LNs was increased with disease but that cells were substantially less phagocytic, as revealed by overall lower levels of payload per cell in TdLNs compared with those in naive LNs (62). These results underscore how, in addition to the antigen-presenting functions of immune cells themselves, the functionality of the lymphatic system as a whole, in combination with the structure of the LN, affects how antigens enter and are processed within the LN.

Strategies have been devised to take advantage of LN remodeling effects on solute and nanoparticle transport to improve drug delivery One such approach has been the delivery of nitric oxide, a reactive small molecule that has immunomodulatory (127) and vasoactive effects on the blood and lymphatic vasculature (128, 129), into lymph. This was achieved by a lymph-draining PPS NP 30 nm in hydrodynamic size engineered to incorporate *S*-nitrosothiols (130). As a result of nitric oxide delivery into lymph, sampling of lymph-borne solutes, including the PPS NP carrier itself as well as coadministered lymph-draining carriers, by LN leukocytes was increased. This approach could thus improve the delivery and immunomodulatory effects of antigen delivered to LN cells, since antigen uptake and presentation remained unaffected by S-nitrosated PPS NP treatment despite improvements in leukocyte access and payload delivery Similarly, ablation of SCS macrophages in LNs, in addition to modulation of collecting lymphatic pump function via administration of a saponin nanovaccine, dramatically enhanced delivery to LN B cells and augmented humoral immunity (131). Depleting SCS macrophages also improves the trafficking of gold nanoparticle–tethered antigen to B cell follicles within LNs from the site of administration, resulting in up to 60× the antibody production (132). Pharmacological inhibition of macrophage uptake function likewise enhanced vaccine efficacy, supporting the idea that altering the SCS macrophage barrier could be an effective strategy to enhance vaccine potency (132).

## CONCLUSION

Given the lymphatic system’s crucial role in the maintenance of tissue fluid homeostasis and adaptive immune response, advances in engineered nanotechnologies for drug delivery have enabled new approaches aiming to leverage the lymphatic system’s therapeutic potential. Targets include lymphatic vessels themselves, LNs and the cells that they contain, and avoidance of first-pass metabolism in the liver. With an increased understanding of lymphatic system physiology and its role in pathophysiology, the role of nanotechnology in enabling lymphatic drug delivery will continue to expand and mature to more translational realization.
